# Delineation of the clinical phenotype associated with non-mosaic type-2 *NF1 *deletions: two case reports

**DOI:** 10.1186/1752-1947-5-577

**Published:** 2011-12-12

**Authors:** Julia Vogt, Rosa Nguyen, Lan Kluwe, Martin Schuhmann, Angelika C Roehl, Tanja Mußotter, David N Cooper, Victor-Felix Mautner, Hildegard Kehrer-Sawatzki

**Affiliations:** 1 Institute of Human Genetics, University of Ulm, Ulm, Germany; 2 Department of Pediatrics, University of Maryland, Baltimore, MD, USA; 3 Department of Maxillofacial Surgery, University Medical Center Hamburg-Eppendorf, Hamburg, Germany; 4 Institute of Medical Genetics, School of Medicine, Cardiff University, Cardiff, UK; 5 Department of Neurosurgery, University of Tübingen, Tübingen, Germany; 6 Department of Neurology, University Medical Center Hamburg-Eppendorf, Hamburg, Germany

## Abstract

**Introduction:**

Large deletions of the *NF1 *gene and its flanking regions are frequently associated with a severe clinical manifestation. Different types of gross *NF1 *deletion have been identified that are distinguishable both by their size and the number of genes included within the deleted regions. Type-1 *NF1 *deletions encompass 1.4 Mb and include 14 genes, whereas the much less common type-2 *NF1 *deletions span 1.2 Mb and contain 13 genes. Genotype-phenotype correlations in patients with large *NF1 *deletions are likely to be influenced by the nature and number of the genes deleted in addition to the *NF1 *gene. Whereas the clinical phenotype associated with type-1 *NF1 *deletions has been well documented, the detailed clinical characterization of patients with non-mosaic type-2 *NF1 *deletions has not so far been reported.

**Case presentation:**

In the present report we characterized two Caucasian European patients with non-mosaic (germline) type-2 *NF1 *deletions. Our first patient was a 13-year-old girl with dysmorphic facial features, mild developmental delay, large hands and feet, hyperflexibility of the joints, macrocephaly and T2 hyperintensities in the brain. A whole-body magnetic resonance imaging scan indicated two internal plexiform neurofibromas. Our second patient was an 18-year-old man who exhibited dysmorphic facial features, developmental delay, learning disability, large hands and feet, hyperflexibility of the joints, macrocephaly and a very high subcutaneous and internal tumor load as measured volumetrically on whole-body magnetic resonance imaging scans. At the age of 18 years, he developed a malignant peripheral nerve sheath tumor and died from secondary complications. Both our patients exhibited cardiovascular malformations.

**Conclusions:**

Our two patients with non-mosaic type-2 *NF1 *deletions exhibited clinical features that have been reported in individuals with germline type-1 *NF1 *deletions. Therefore, a severe disease manifestation is not confined to only patients with type-1 *NF1 *deletions but may also occur in individuals with type-2 *NF1 *deletions. Our findings support the concept of an *NF1 *microdeletion syndrome with severe clinical manifestation that is caused by type-1 as well as type-2 *NF1 *deletions.

## Introduction

Neurofibromatosis type I (NF1) is an autosomal-dominant inherited disease with an estimated frequency of 1:3000 [1]. The classic hallmarks of NF1 include café-au-lait spots, axillary and inguinal freckling, neurofibromas and Lisch nodules. Approximately 95% of all patients with NF1 harbor mutations within the *NF1 *gene, whereas 5% have large deletions that encompass the *NF1 *gene and its flanking regions [2]. From a clinical point of view, the molecular diagnosis of a large *NF1 *deletion is important because they are frequently associated with severe clinical manifestations [3-6] including an increased risk of malignant peripheral nerve sheath tumors (MPNSTs) as compared with the general NF1 population [7]. MPNSTs and gliomas together represent the most common causes of reduced life expectancy among patients with NF1 [8].

**Figure 1:**
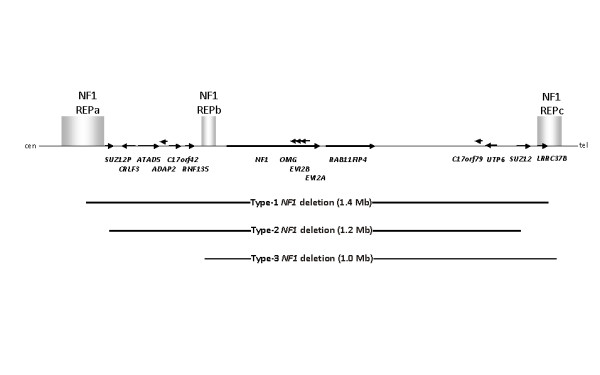
**Schema of the *NF1 *gene region and relative extents of the different deletion types**. The 14 genes located within the *NF1 *gene region, and included within the type-1 deletion region, are indicated by horizontal arrows. The direction of each arrow denotes the transcriptional orientation of the corresponding gene. The relative locations of the low-copy repeats, termed NF1-REPa, NF1-REPb and NF1-REPc are also indicated by vertical bars. Cen, centromeric; tel, telomeric.

Three types of recurrent large *NF1 *deletion have so far been reported: the type-1 deletion, present in 70% to 80% of all patients with NF1 with large *NF1 *deletions, is the most frequent [6,9]. The breakpoints of type-1 *NF1 *deletions are located within low-copy repeats, termed NF1-REPa and NF1-REPc [10]. Type-1 deletions encompass 1.4 Mb and lead to the loss of 14 genes (Figure 1). By contrast, type-2 *NF1 *deletions span 1.2 Mb and include only 13 genes since *LRRC37B *is not deleted (Figure 1). The breakpoints of type-2 deletions are located within *SUZ12 *and its pseudogene *SUZ12P *[11-13]. With an estimated frequency of 9% to 20%, type-2 deletions are less frequent than type-1 *NF1 *deletions [6,9,12]. A third type of recurrent *NF1 *deletion (type-3) has recently been reported which encompass 1.0 Mb and include nine genes (Figure 1) [6,14]. In addition to these three types of recurrent *NF1 *deletion mediated by non-allelic homologous recombination (NAHR), an amorphous group of atypical *NF1 *deletions has also been observed in a subset of patients. These atypical *NF1 *deletions are characterized by non-recurrent breakpoints and they are highly heterogeneous in terms of their size, breakpoint position and the number of genes present within the deleted region [6,9].

It is widely assumed that genotype-phenotype correlations in patients with large *NF1 *deletions are likely to be influenced by both the number and type of deleted genes. The comprehensive clinical characterization of 29 patients with NF1 with molecularly ascertained type-1 *NF1 *deletions identified several features that are frequently associated with this type of deletion [5]. These features include dysmorphic facial features, tall stature, large hands and feet, scoliosis, developmental delay, learning disabilities and a high burden of subcutaneous, plexiform and spinal neurofibromas.

In contrast to the well documented clinical phenotype associated with type-1 *NF1 *deletions, the detailed clinical investigation of patients with non-mosaic type-2 *NF1 *deletion has not been published so far. Type-2 deletions frequently arise during post-zygotic cell division and consequently give rise to somatic mosaicism with normal cells alongside those cells harboring the deletion [11-13]. At least 44% of all patients with NF1 with type-2 *NF1 *deletions exhibit somatic mosaicism [9] but their prevalence may well be under-estimated [12]. Studies of genotype-phenotype correlations in patients with mosaic type-2 *NF1 *deletions are inherently difficult to perform because the number of normal cells lacking the deletion in different tissues (and hence their impact on the clinical phenotype) is difficult to assess. Somatic mosaicism is very likely to exert a major influence on disease severity since patients with mosaic large *NF1 *deletions often display a mild clinical phenotype [11-13]. Whereas somatic mosaicism is an issue that needs to be addressed in all cases of type-2 *NF1 *deletion, somatic mosaicism is rare among patients with type-1 *NF1 *deletions, occurring in only 2% to 4% of cases [9].

In order to assess the phenotype associated with type-2 *NF1 *deletions, careful clinical examination of patients with non-mosaic (germline) type-2 *NF1 *deletions is critically important. However, germline type-2 *NF1 *deletions are rare, with an estimated frequency of < 6% among patients with NF1 with gross *NF1 *gene deletions [9]. In a previous study, we specifically sought type-2 *NF1 *deletions among patients with *NF1 *deletion in order to explore the causative mutational mechanisms underlying these deletions [13]. We succeeded in identifying 18 patients with type-2 deletions, 16 of whom exhibited somatic mosaicism with normal cells. The remaining two patients with type-2 *NF1 *deletions in whom mosaicism could not be detected were considered to be probable germline type-2 deletions.

In the present study, we describe the clinical features of two patients with non-mosaic type-2 *NF1 *deletions and compare their phenotypes to those of patients with type-1 *NF1 *deletions. Our aim was to assess whether obvious differences exist in relation to the clinical phenotypes associated with these two different types of *NF1 *deletion.

## Case presentation

The characterization of the deletion breakpoints of both patients (2429 and 2358 in [13]) studied here has been reported previously [13]. Breakpoint analysis indicated that the deletions were mediated by NAHR between the *SUZ12 *gene and *SUZ12P*. Neither FISH performed on blood samples nor microsatellite marker analysis using DNA from buccal swabs yielded any evidence for somatic mosaicism with normal cells in both patients [13]. In our first patient (patient 2358 in [13]), FISH was performed on interphase cells derived from an urine sample according to the methods described previously [12]; of 307 nuclei evaluated > 99% exhibited the deletion. We conclude that it is likely that both patients harbor non-mosaic (germline) type-2 *NF1 *deletions.

### Patient 1

**Figure 2:**
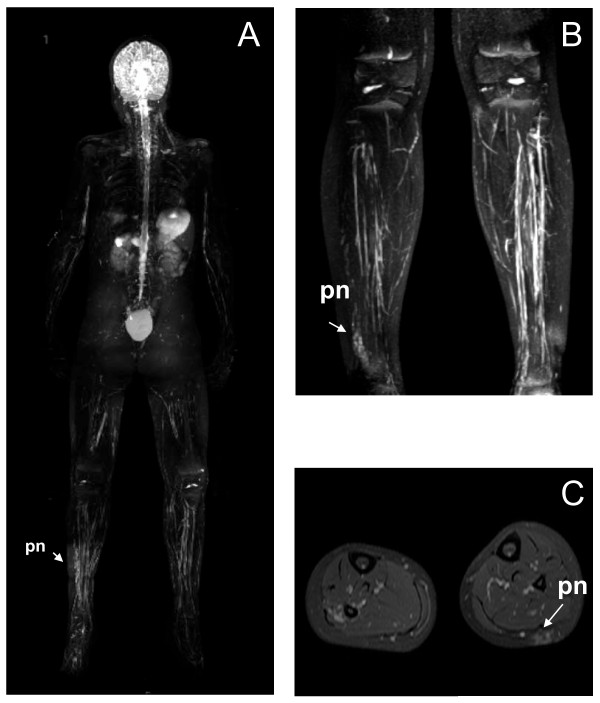
**Whole body MRI of our first patient indicated a superficial plexiform neurofibroma (pn) of the left calf (A, B) and an internal plexiform neurofibroma above the right ankle (C)**.

Our first patient was a prematurely born girl of Caucasian European descent, with a patent foramen ovale with a minor left-to-right cardiac shunt and pulmonary valve stenosis (PVS) at birth. However, the PVS diminished over time. During her pre-school years, she exhibited mild motor and cognitive developmental delay. At the age of five years, delays in speech and the development of articulation were diagnosed. Clinical examination at the age of 12 years and nine months indicated normal height and weight (160 cm (50th to 75th percentile) and 59 kg (75th to 90th percentile)). However, macrocephaly was noted with a head circumference of 56.8 cm (97th percentile). Facial dysmorphic features including hypertelorism, epicanthic folds, a broad nasal root and low set ears were observed. She had large hands and feet and hyperflexibility of the joints. More than six café-au-lait spots were noted as well as axillary and inguinal freckling, Lisch nodules and 10 subcutaneous neurofibromas. She had slight hypertrichosis on her lower back and a length difference of the legs. Neurological examination indicated generalized muscular hypotonia, but she showed adequate coordination and motor function. Whole body MRI revealed a small plexiform neurofibroma of the right calf (9.2 mL, Figure 2A, B) and an internal plexiform neurofibroma above the right ankle (Figure 2C). Neither large occult tumors, nor spinal neurofibromas were identified on whole-body MRI scans (Figure 2A). MRI of the brain revealed T2 hyperintensities in the cerebellum, which are thought to represent foci of neural dysplasia or dysmyelination. Neuropsychological testing was not performed. Initially, our patient attended a regular elementary school and then, at the age of 12 years and nine months, secondary school. Severe learning disabilities were not reported, but owing to attention deficit and impairment in speed of learning, she needed educational support.

### Patient 2

**Figure 3:**
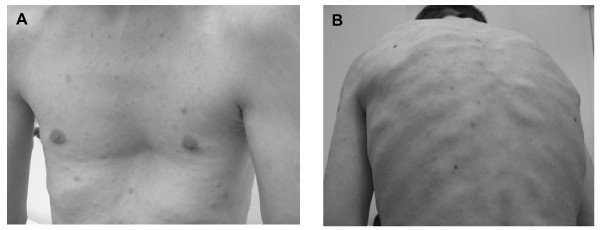
**(A) Funnel chest and cutaneous neurofibromas on the trunk of our second patient, and multiple subcutaneous neurofibromas on the back of our patient (B)**.

Our second patient was a man of Caucasian European descent who had shown considerable motor and cognitive developmental delay as a child in his pre-school years. He was clinically examined by us at the age of 18 years. His body measures were within the normal range (height: 184 cm (50th to 75th percentile), weight: 62 kg (10th to 25th percentile)). However, macrocephaly was noted (head circumference: 60 cm (97th percentile)). He had large hands and feet and hyperflexibility of the joints. Facial dysmorphic features including saddle nose and hypertelorism were noted, as well as a broad neck and a funnel chest (Figure 3A). A difference in length between his legs was noted but his spine was in an orthograde position. MRI of his brain revealed complex corpus callosum aplasia. Neuropsychological examination revealed significant visual motor and attention deficits. IQ testing indicated a full scale IQ of 84, a verbal IQ of 86 and a performance IQ of 85. Our patient attended regular elementary school and obtained a secondary school certificate.

**Figure 4:**
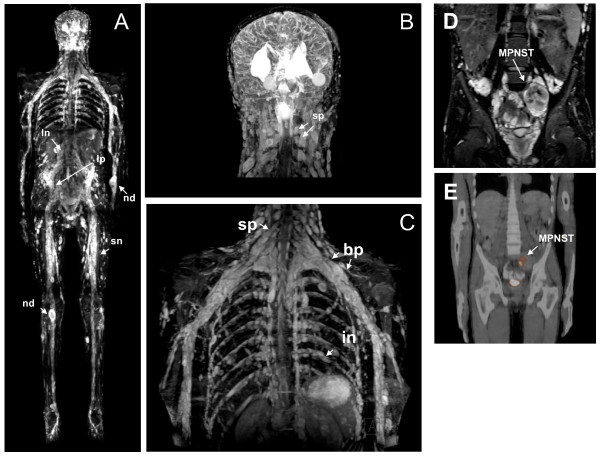
Internal tumor load of our second patient. Whole body MRI **(A)** indicated multiple tumors along the lumbar nerves (ln), the sciatic nerves (sn) and within the lumbar plexus (lp). Nodular neurofibromas (nd) were also detected. A coronal image of his head **(B)** indicated multiple spinal neurofibromas (sp), which were also detected on MRI images of his trunk **(C)** as well as neurofibromas within the brachial plexus (bp) and along the intercostal nerves (in). MRI also showed a high tumor density in the lesser pelvis of our patient **(D)**, including a malignant peripheral nerve sheath tumor (MPNST) as confirmed by positron emission tomography - computed tomography (PET-CT) **(E)**.

He had more than six café-au-lait spots and axillary and inguinal freckling as well as Lisch nodules. Cardiac examination showed a right ventricular subvalvular neurofibroma (17 × 17 mm), which resulted in a first-degree pulmonary valve insufficiency. More than 1000 subcutaneous and 500 cutaneous neurofibromas were noted (Figure 3B). Volumetric analysis of whole body MRI scans indicated a high internal total tumor load of 3896 mL. Large plexiform neurofibromas (PNs) were detected along the lumbo-sacral plexus and in the abdomen/pelvis. Smaller PNs were detected within the right lower limb, parapharyngeal space, along the brachial plexus and spinal nerves as well as major peripheral nerves (Figure 4A-C).

Our patient's primary MPNST (WHO grade IV) evolved from a pre-existing PN located in the lesser pelvis affecting the lumbar vertebra L5 and leading to osteolysis of his sacral and ileac bone. Seven months after surgery, our patient experienced severe abdominal pain and relapse was evident on MRI and PET-CT scan (standardized uptake value (SUV) of 11,5, Figure 4D, E). Our patient underwent surgery and adjuvant chemotherapy (CWS protocol) but post-operative staging revealed tumor infiltration of the intestine up to the left colon flexure and hence, maintenance chemotherapy (CWS-2002-P study) and radiation therapy were initiated. Three weeks before he died, our patient and his relatives opted to terminate the maintenance therapy.

## Discussion

Comparison of the clinical features of our two patients with non-mosaic type-2 *NF1 *deletions reported here with the manifestations of disease in patients with molecularly ascertained type-1 deletions reported by Mautner *et al*., [5] indicated a considerable overlap of their clinical phenotypes. Among the most frequent clinical features observed in patients with type-1 *NF1 *deletions were dysmorphic facial features, macrocephaly, hyperflexibility of the joints, large hands and feet, cognitive impairment/learning disability, and a high tumor load as well as an increased risk of MPNSTs (Table 1). These features were also noted in our second patient. He had an extremely high internal tumor load (Figure 4), indicative of the severe disease manifestations resulting from the type-2 *NF1 *deletion. Our patient died of the complications associated with a secondary, therapy-resistant MPNST at the age of 18 years. Patients with large *NF1 *deletions have been reported to have a substantially higher risk of development of MPNSTs as compared with NF1 individuals lacking large *NF1 *deletions. Whereas the lifetime risk of an MPNST in all patients with NF1 is 8% to 13%, patients with *NF1 *deletion have an estimated lifetime risk of an MPNST of 16% to 26% [7]. In the study of Mautner *et al*. [5], 21% of the patients with molecularly ascertained type-1 *NF1 *deletions had an MPNST, thereby confirming the increased risk with respect to this malignant tumor experienced by patients with type-1 *NF1 *deletions. Our study suggests that patients with type-2 *NF1 *deletions may also be subject to an increased risk of MPNSTs. Consequently, patients with this type of deletion should also receive intensive medical care in order to guarantee early detection of the malignant transformation of tumors.

**Table 1:** Comparison of the prevalence of clinical features observed in patients with type 1 *NF1 *deletions as reported by Mautner *et al*. [5] with the clinical features noted in our two patients with non-mosaic type-2 *NF1 *deletions

Clinical features of patients with type-1 *NF1 *deletions (percentage of patients with this feature)^a^	Presence or absence of this feature in our patients by breakpoint	
	
	Patient 2429	Patient 2358
Facial dysmorphism (90%)	+	+
Tall stature (46%)^b^	-	-
Large hands and feet (46%)	+	+
Macrocephaly (39%)^c^	+	+
Learning disabilities (48%)	+	+ (mild)
Attention deficits (33%)	+	+
Scoliosis (43%)	-	NA
Hyperflexibility of the joints (72%)	+	+
Plexiform neurofibromas (76%)	+ (multiple)	+
Subcutaneous neurofibromas (76%)	+ (multiple)	+
Cutaneous neurofibromas (86%)	+ (multiple)	-
MPNST (21%)	+	-
T2 hyperintensities (45%)	-	+
Muscular hypotonia (45%)	NA	+
Congenital heart defects (21%)	+	+

^a^According to the analysis of 29 patients with type-1 *NF1 *deletion [5].

^b^Tall stature was denoted if height measurements were at or above the 94th percentile.

^c^Macrocephaly was denoted if the head circumference was at or above the 97th percentile.

-, absent; +, present; MPNST, malignant peripheral nerve sheath tumor; NA, not assessed.

The clinical phenotype observed in our first patient was milder than the phenotype observed in our second patient. However, our first patient was only 13 years old at the time of examination and therefore the load of age-dependent symptoms (such as number of neurofibromas) cannot yet be fully assessed. Furthermore, NF1 is generally associated with high variability in terms of its clinical expression. Hence, some degree of variability in clinical expression is to be expected in patients with large deletions. Regardless, our first patient also exhibited several symptoms known to be associated with type-1 *NF1 *deletions such as dysmorphic facial features, macrocephaly, large hands and feet, hyperflexibility of the joints, mild motor and cognitive developmental delay and T2 hyperintensities. Additionally, she had a congenital heart defect (patent foramen ovale). Congenital heart defects certainly belong to the *NF1 *deletion-associated phenotype as they were also observed in 21% of patients with type-1 *NF1 *deletion [5]. Our second patient also had a pulmonary valve insufficiency (grade I).

The clinical phenotype observed in our two patients with putative germline type-2 *NF1 *deletions reported in this study is broadly similar to those associated with type-1 *NF1 *deletions [5,6]. Also in one of our previous studies, severe clinical phenotypes were observed in two patients with germline type-2 *NF1 *deletions [11]. However, the patients reported in that study could not be investigated by us in great detail with respect to their clinical manifestations; therefore, further conclusions were not possible [11].

The severe clinical phenotype often associated with large *NF1 *deletions has been suggested to represent a contiguous gene syndrome [6]. Accordingly, the clinical manifestations of the disease are likely to be influenced not only by the deletion of the *NF1 *gene but also by the deletion of flanking genes. In contrast to type-1 deletions, the *LRRC37B *gene of unknown function is not included within the deletion interval of type-2 deletions (Figure 1). As yet, it is unclear whether haploinsufficiency for the *LRRC37B *gene exerts any influence on the clinical phenotype associated with large *NF1 *deletions. In this context, it seems worthwhile to note that our two patients clinically investigated in this study did not exhibit severe mental retardation. In the study of Mautner *et al*. [5], 38% of patients with molecularly assigned type-1 *NF1 *deletions exhibited severe mental retardation. Further clinical investigations of additional patients with NF1 with non-mosaic type-2 *NF1 *deletions will be necessary in order to assess whether severe mental retardation is a feature that occurs more often in association with type-1 than type-2 *NF1 *deletions.

## Conclusions

The clinical phenotype of patients with non-mosaic type-2 *NF1 *deletions can be severe and includes most of the features that have been reported in patients with type-1 *NF1 *deletions. Our findings support the existence of a *NF1 *microdeletion syndrome caused by type-1 as well as type-2 *NF1 *deletions. The additional complications associated with the *NF1 *microdeletion syndrome render the intensive clinical and psychological care of patients who are affected absolutely necessary.

## Consent

Written informed consent was obtained from the legal guardians/parents of both patients for publication of this case report and any accompanying images. A copy of the written consent is available for review by the Editor-in-Chief of this journal.

## Competing interests

The authors declare that they have no competing interests.

## Authors' contributions

JV, HK-S and DNC wrote the manuscript; RN, LK and V-FM performed the clinical investigations; ACR, JV and TM performed the mutation analysis. All authors read and approved the final manuscript.
